# Sonographically determined kidney measurements are better able to predict histological changes and a low CKD-EPI eGFR when weighted towards cortical echogenicity

**DOI:** 10.1186/s12882-020-01789-7

**Published:** 2020-04-06

**Authors:** Nordeval Cavalcante Araújo, Maria Alice Puga Rebelo, Lilimar da Silveira Rioja, José Hermógenes Rocco Suassuna

**Affiliations:** 1 Nephrology section, University of the State of Rio de Janeiro, Boulevard 28 de Setembro, 77 – Vila Isabel, Rio de Janeiro, RJ 20551-030 Brazil; 2 Pathology section, University of the State of Rio de Janeiro, Rio de Janeiro, Brazil

**Keywords:** Renal sonography, Renal biopsy, Cortical echogenicity, Renal length, Cortical thickness

## Abstract

**Background:**

The renal length and cortical echogenicity have shown correlation to the renal function and histological changes in CKD patients. The aim of this study was to assess the accuracy of crude and composite ultrasound parameters based on kidney measurements and cortical echogenicity to detect renal dysfunction and histological changes.

**Methods:**

Kidney sonography and biopsy were performed in 112 patients. Histological changes were graded in 0, < 25%, ≥25%, ≤50 and > 50% of the sample. Cortical echogenicity was graded relative to liver or spleen parenchyma: less than, equal to and higher than the liver/spleen. Kidney length, the kidney length/body height ratio (KL/H) and cortical thickness were obtained. Each parameter was multiplied by a cortical echogenicity-weighting arbitrary factor: 1.17, 1 or 0.69 for cortex less than, equal to or higher than the liver, respectively. The GFR was estimated using the CKD-EPI formula. The accuracy of crude and composite parameters to identify patients with a high creatinine, a low GFR and histological changes were evaluated.

**Results:**

The discriminative power of kidney length and cortical thickness for renal dysfunction and histological changes was improved after weighting for cortical echogenicity. However, the best discriminative was the kidney length to height ratio weighted towards renal echogenicity (w-KL/H).

**Conclusion:**

w-KL/H exceeded the other parameters as a marker of renal impairment and histological changes in CKD. Calculation of the w-KL/H index may be of help as a non-invasive tool to identify patients with significant renal disease and might be useful to guide therapeutic decisions.

## Background

Renal ultrasound is usually the first imaging procedure used in the clinical assessment of patients with renal disease [1]. Some sonographically determined kidney parameters correlate with renal function, including kidney length (KL) [2, 3] and cortical echogenicity, which is the most commonly reported parameter at renal ultrasound [4]. Additionally, changes in kidney length/body height ratio (KL/H) [5] and cortical thickness (CT) [6] are believed to show a relationship with renal function in chronic kidney disease (CKD) patients.

The assessment of relative hepatorenal echogenicity has been used in determining cortical echogenicity in the renal parenchymal evaluation. Increased cortical echogenicity is a marker of renal disease that correlates to severity of interstitial histological changes in renal parenchymal disease [5]. Normally, the renal cortical echoes are lower in amplitude than either the normal parenchyma of the spleen or the liver [7]. However, a study claimed that in a percentage (around 33.0%) of normal right kidneys the echogenicity equaled that of the liver [8]. On the other hand, in adults, a renal cortex more echogenic than the liver is clearly abnormal and indicates renal disease [9].

Cortical thickness has been claimed to be better than renal length as an indicator of renal function in chronic kidney disease [6]. However, despite the fact that renal length is the most commonly used parameter of renal assemble in the clinical setting, the kidney length to body height ratio better represents kidney size than the absolute renal length [10]. Therefore, its use in chronic renal disease studies would be valuable.

Accordingly, we evaluated the performance of the crude values of renal size, renal length/height ratio and cortical thickness and after multiplication by a derived cortical echogenicity-weighting factor. The purpose of our study was to assess the ability of these parameters to identify patients with a serum creatinine > 1.5 mg/dl and with an eGFR ≤45 ml/min and severe histological changes on biopsy samples.

## Methods

This study was approved by the local ethics committee. All participants provided written informed consent.

Kidney sonographic examinations were performed in 118 patients, immediately prior to percutaneous renal biopsy, in patients with proteinuria, hematuria and decreased renal function. Primary and secondary renal diseases were included. Additionally, a pathology report for diagnosis purposes, including data on glomerular obsolescence (GO), mesangial proliferation (MP), glomerular cellularity (GC), crescent and fibrinoid necrosis (CFN), glomerular sclerosis (GS), tubular atrophy (TA), interstitial fibrosis (IF) and interstitial infiltrate (II) were graded according to extension and severity in 0 (0%, no change), 1 (< 25% of the sample), 2 (≥25% ≤ 50% of the sample), and 3 (> 50% of the sample). All ultrasounds were performed by the same operator (NCA) and all biopsies were evaluated by the same pathologist (LSR).

Recently, a standardized grading of chronic changes in native renal biopsy samples was proposed based on four levels (0 to 3) for glomerulosclerosis (GS), tubular atrophy (TA) and interstitial fibrosis (IF) and two levels (0 to 1) for arteriosclerosis. The score is calculated by summing the four pathologic changes levels and ranges from 0 to 10, with a cut-off value > 8 established for severe chronic changes [11]. In our study, using a modification of this method, an index was constructed based on four levels (0 o 3) for GS, TA and IF. Since arteriosclerosis was not included in the index, a value of > 7 was set as the cut-off for severe chronic changes.

**Fig. 1:**
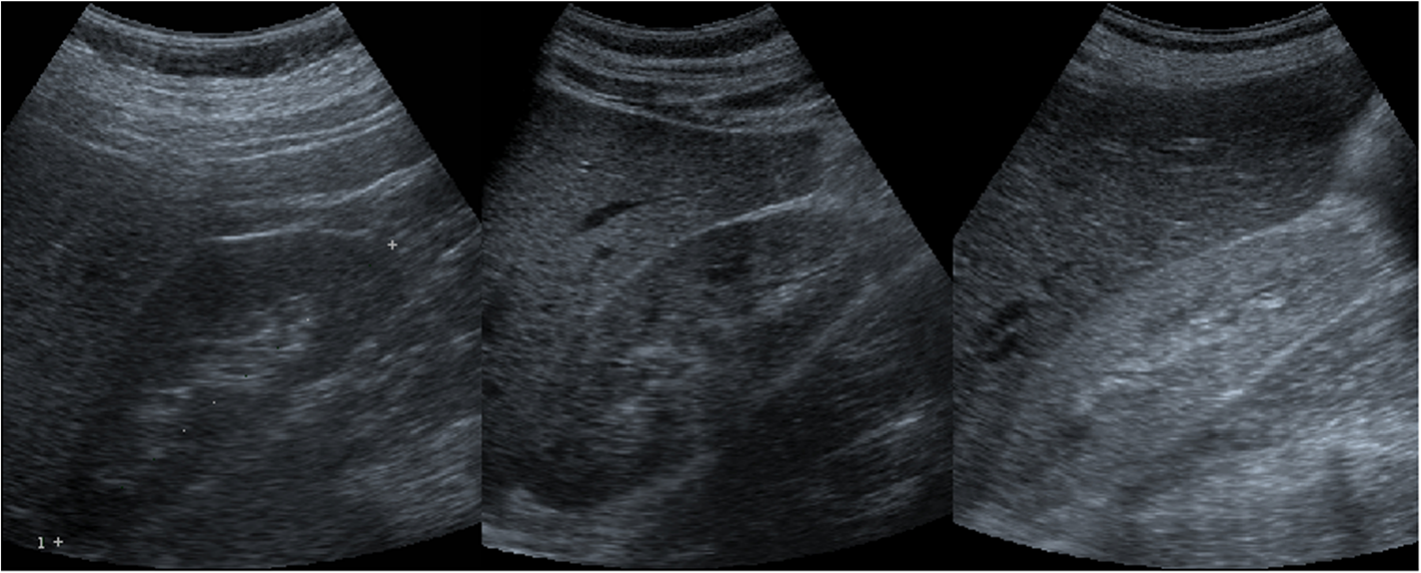
Sonograms were graded by the degree of cortical echogenicity relative to the liver or spleen parenchyma in the following manner: Left: less than (grade 0); Middle: equal to (grade 1); Right: greater than (grade 2)

**Fig. 2:**
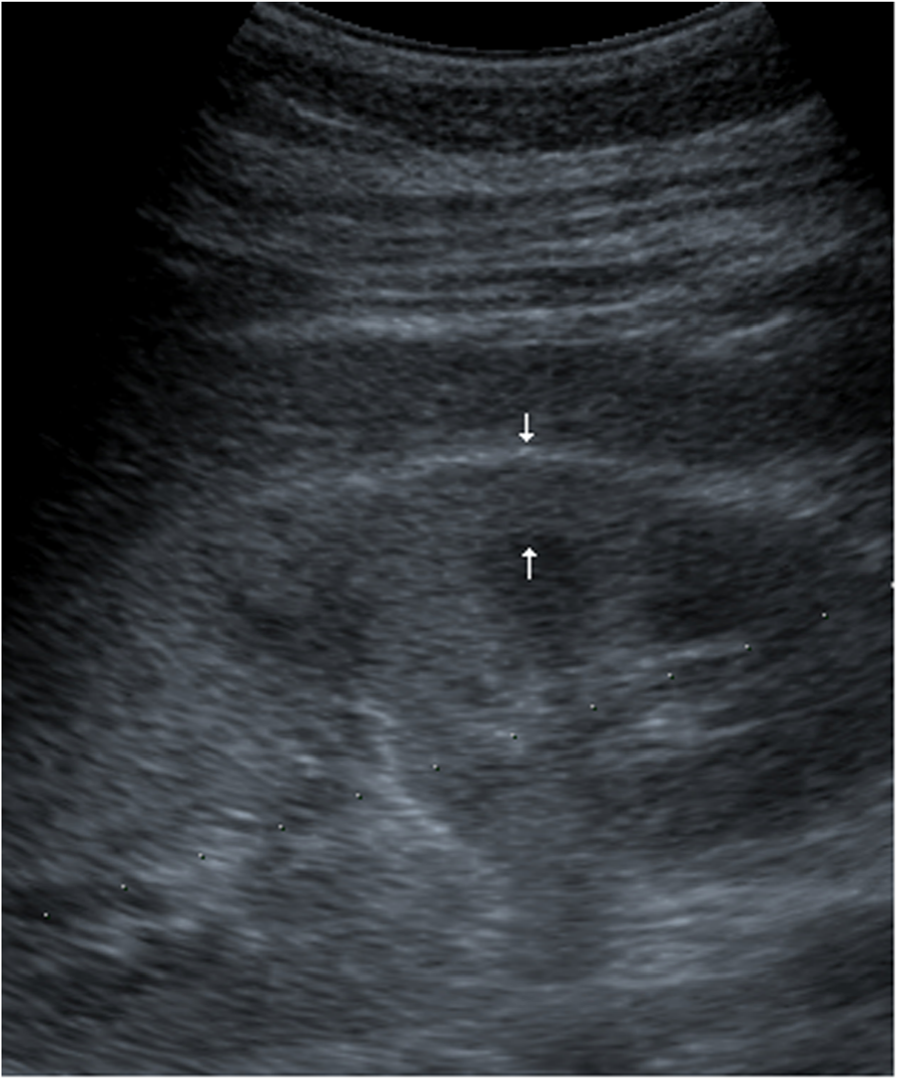
The measurement of cortical thickness was made from the outer renal cortical margin to the outer margin of the medullary pyramid (arrows), in the coronal plane

The ultrasound studies were performed using a commercially available unit with a 3.5 MHz transducer (Sonoline 40, Siemens, Erlangen, Germany). Renal parenchyma was graded by the degree of cortical echogenicity relative to the liver/spleen parenchyma: less than, equal to and higher than the liver or spleen (Fig. 1). Values of ultrasound parameters such as kidney length (KL), the kidney length (mm)/body height (cm) ratio (KL/H) and cortical thickness (CT) were obtained. Kidney length was measured as the maximal longitudinal dimension as visualized in the longitudinal plane parallel to the longest renal axis. The measurement of the cortical thickness was made from the outer renal cortical margin to the base of a medullary pyramid in the coronal plane (Fig. 2).

The glomerular filtration rate (GFR) was estimated from the serum creatinine (SCr) concentration, performed within 30 days of the ultrasound, using the CKD-EPI (Chronic Kidney Disease Epidemiology Collaboration) formula: GFR = 141 x min(Scr/κ,1)α x max(Scr/κ,1)-1.209 × 0.993Age × 1.018 [if female] × 1.159 [if black] [12].

We calculated the areas under the receiver operator characteristic curves (AUROC) to evaluate the ability of ultrasound parameters to identify patients with a high SCr (> 1.5 mg/dl) and a low eGFR (≤ 45 ml/min). Additionally, the same procedure was used to evaluate the discriminative power of every parameter (SCr, CKD-EPI and ultrasound parameters) to identify the severity and extension of histological changes and the index of severe chronic changes (score > 7).

Next, we re-evaluated the performance of the ultrasound parameters with ROC curve analysis after multiplication by an empirically-derived semi-quantitative cortical echogenicity-weighting arbitrary factor (w). To create the weighting factor, we used the range from 0.71 to 1.89 for renal cortical/ liver echogenicity ratio reported by others [13] as the grading scale. For a renal cortex equal to the liver in echogenicity, a factor of 1 was assigned. For cortical echogenicity less than and greater than the liver the inverse of an intermediate value between 0.71 and 1 (1.17) and between 1 and 1.89 (0.69) was chosen, respectively. The reasoning for using an inverse value is that this procedure renders it more appropriate for use as a weighting factor, i.e. it points in the same direction (disease severity) as the main parameter such that is it higher if the renal cortex is less echogenic than the liver (less severe disease) and lower in the opposite case (more severe disease).

Accordingly, ROC curve analysis was performed to evaluate the ability of weighting ultrasound parameters (w-CT, w-KL, w-KL/H) to identify patients with a high serum creatinine (> 1.5 mg/dl) and a low eGFR (≤45 ml/min) and to predict a chronic histological index > 7.

Significance was considered at a p value <  0.05.

## Results

Right kidney cortical echogenicity relative to liver echogenicity was obtained in all patients, except in six whose liver was fatty. In these cases, the left cortical kidney echogenicity relative to the spleen was considered. Six out of 118 patients with no representative biopsy sample were excluded. Therefore, 112 patients were enrolled in the study.

Patient characteristics and clinical data are shown in Table 1. The patients ranged in age from 13 to 76 years at the time of biopsy (mean ± standard deviation age 36.4 ± 16.1 years); there were 61 females and 76 Caucasians. The mean ± standard deviation of serum creatinine level was 2.08 ± 1.96 mg/dl (range 0.60 to 11.7 mg/dl) and of CKD-EPI was 67.5 ± 44.8 ml/min (range 1.0 to 163.0 ml/min). In total, 46 patients had impaired renal function (Cr >  1.5 mg/dl). The crude and weighted for cortical echogenicity ultrasound parameters are shown in Table 1.

**Table 1:** Baseline demographics and clinical characteristics of the patients

Number of patients	112
Male/Female	51/61
Caucasian/African Brazilian	76/36
Age (years)	36.4 ± 16.1
Height (cm)	164.0 ± 9.8
Weight (kg)	66.3 ± 16.9
Serum Creatinine (mg/dL)	2.08 ± 1.96
CKD-EPI GFR (ml/min)	67.5 ± 44.8
24-h urine protein (g/day)	6.10 ± 5.90
Cortical Thickness (mm)	8.78 ± 1.82
Kidney Length (mm)	109.0 ± 11.0
Kidney Length/Height (mm/cm)	0.67 ± 0.06
w-Cortical Thickness	8.71 ± 2.65
w-Kidney Length	106.5 ± 25.0
w-Kidney Length/Height	0.66 ± 0.15

*CKD-EPI* chronic kidney disease epidemiology collaboration, *GFR* glomerular filtration rate, *w* weighting for cortical echogenicity

**Fig. 3:**
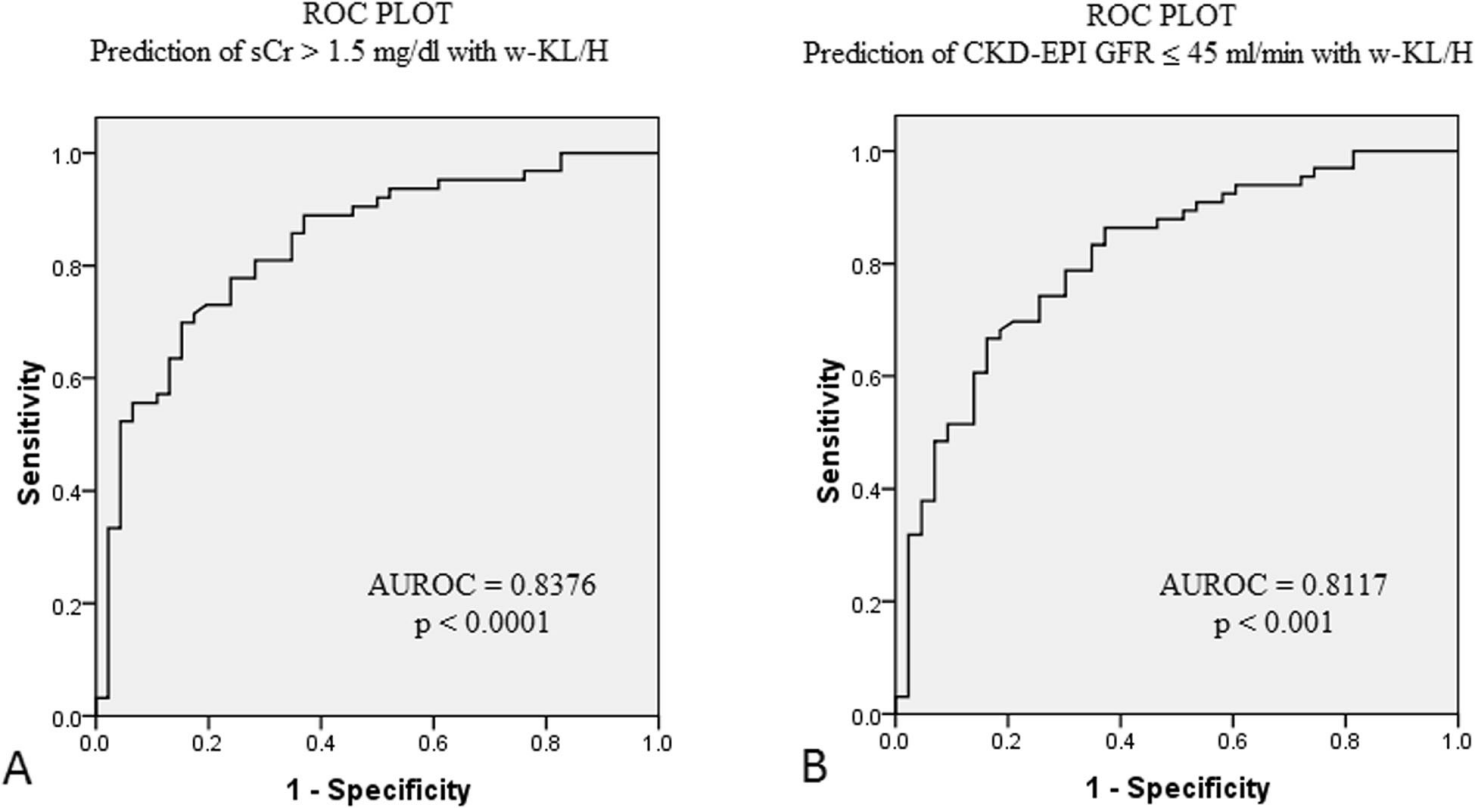
Receiver operating characteristic curve to evaluate the diagnostic accuracy of w-KL/H to predict increased serum creatinine and low CKD-EPI

Crude KL/H performed better than kidney length and cortical thickness for the detection of increased serum creatinine and decreased CKD-EPI (Table 2). However, the ability of absolute parameters to identify patients with a low eGFR or an abnormal serum creatinine was marginal. Discriminative power was markedly improved after weighting for cortical echogenicity (Table 2). The best discriminative parameter (at the cut-off <  0.66) was the newly derived index: the kidney length to height ratio weighted towards relative renal echogenicity (w-KL/H) (Table 2 and Fig. 3).

**Table 2:** Receiver operating characteristic (ROC) analysis of crude and weighted sonographic parameters in relation to their ability to identify patients with significant renal dysfunction

	Patients meeting criteria	ROC Analysis	CT	KL	KL/H	w-CT	w-KL	w-KL/H
SCr > 1.5 mg/dL	41.1%	AUROC p	0.617 0.051	0.565 0.243	0.619 0.033	0.776 < 0.001	0.806 < 0.001	0.838 < 0.001
CKD-EPI eGFR < 45 ml/min	40.2%	AUROC p	0.607 0.076	0.604 0.064	0.639 0.014	0.754 < 0.001	0.793 < 0.001	0.812 < 0.001

*AUROC* area under the receiver operating characteristic, *KL* kidney length, *H* height, *CT* cortical thickness, *w* weighted, *SCr* serum creatinine, *eGFR* estimated glomerular filtration rate, *CKD-EPI* chronic kidney disease epidemiology collaboration

Table 3 shows the accuracy of the area under the ROC for significant renal dysfunction defined as serum creatinine >  1.5 mg/dl or CKD-EPI < 45 ml/min and crude and weighted ultrasound parameters to detect semi-quantitatively graded renal histological changes. For every chronic glomerular and interstitial lesion examined, w-KL/H performed better than SCr and CKD-EPI and all crude and weighted ultrasound parameters. None of parameters were able to detect acute glomerular changes.

**Table 3:** Receiver operating characteristic (ROC) analysis of the relationship of significant renal dysfunction and crude and weighted for cortical echogenicity ultrasound parameters to semi-quantitatively graded renal histological changes

	ROC Analysis	MP	GC/N	GC	GS	GO	TA	IF	II
SCr > 1.5 mg/dL	AUROC p	0.503 0.952	0.503 0.964	0.532 0.563	0.623 0.027	0.676 0.002	0.621 0.030	0.656 0.005	0.706 < 0.001
CKD-EPI < 45 ml/min	AUROC p	0.517 0.761	0.509 0.870	0.542 0.460	0.627 0.024	0.683 0.001	0.624 0.028	0.679 0.001	0.710 < 0.001
CT < 8.78 mm	AUROC p	0.516 0.785	0.578 0.198	0.533 0.591	0.476 0.694	0.608 0.075	0.530 0.620	0.580 0.188	0.555 0.366
KL < 109 mm	AUROC p	0.457 0.436	0.480 0.719	0.480 0.724	0.528 0.613	0.656 0.005	0.593 0.093	0.542 0.447	0.511 0.839
KL/H < 0.67	AUROC p	0.393 0.054	0.476 0.671	0.477 0.680	0.636 0.015	0.631 0.019	0.640 0.012	0.630 0.019	0.557 0.304
w-CT < 8.71	AUROC p	0.510 0.873	0.555 0.361	0.535 0.558	0.559 0.327	0.583 0.167	0.627 0.035	0.665 0.006	0.648 0.014
w-KL < 106.5	AUROC p	0.424 0.183	0.492 0.893	0.478 0.700	0.656 0.006	0.657 0.006	0.694 0.001	0.685 0.001	0.687 0.001
w-KL/H < 0.66	AUROC p	0.458 0.463	0.535 0.542	0.516 0.775	0.736 < 0.001	0.693 0.001	0.716 < 0.001	0.714 < 0.001	0.766 < 0.001

*MP* mesangial proliferation, *GC/N* glomerular crescents/necrosis, *GC* glomerular celularity, *GS* glomerular sclerosis, *GO* glomerular obsolescence, *TA* tubular atrophy, *IF* interstitial fibrosis, *II* interstitial infiltrate, *SCr* serum creatinine, *AUROC* area under the receiver operating characteristic, *CKD-EPI* chronic kidney disease epidemiology collaboration, *CT* cortical thickness, *KL* kidney length, *KL/H* kidney length/height, *w* weighted for cortical echogenicity factor

**Fig. 4:**
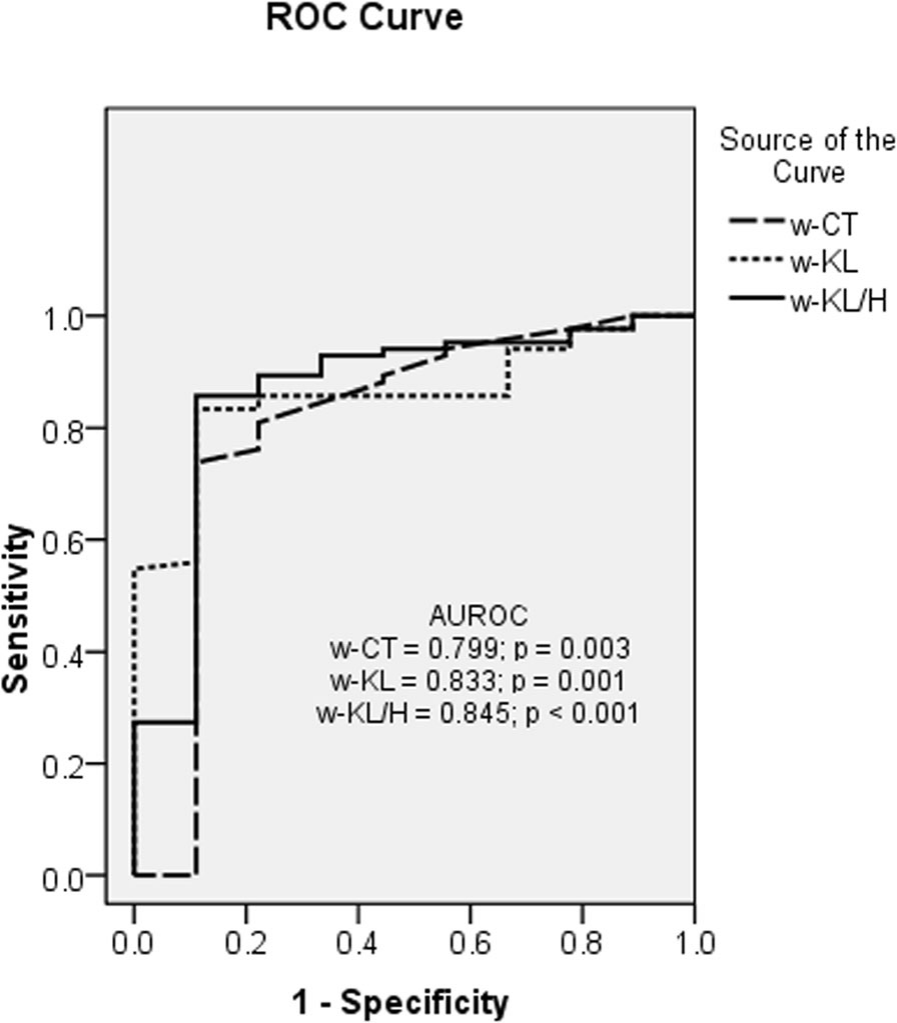
Receiver operating characteristic curve to evaluate the diagnostic accuracy of weighted (w) for cortical echogenicity cortical thickness (w-CT), kidney length (w-KL) and the kidney length/height ratio (w-KL/H) to predict severe grades of chronic histological changes (glomerular sclerosis, tubular atrophy and interstitial fibrosis) based on a total score > 7

In Table 4, the performance of every single parameter (renal function or ultrasound) for detection of index of chronic changes (score of > 7) is shown. Based on the simple inspection of values, without statistical analysis, among the weighted ultrasound parameters, w-KL/H had the best performance (Fig. 4). Instead of data analysis using the Youden test, the cut-off was chosen based on a decision-analytic approach (utility based) [14] for a sensitivity as close as possible to 95%.

**Table 4:** Area under the curve to predict the diagnostic accuracy of parameters studied for severe grades of chronic histological changes (glomerular sclerosis, tubular atrophy and interstitial fibrosis) based on total score > 7

Variable	AUROC	p	Cut-off	Sensitivity	Specificity
SCr	0.703	0.035	7.5	90.0%	20.0%
CKD-EPI	0.728	0.018	11.5	94.0%	40.0%
CT	0.599	0.331	–	–	–
KL	0.607	0.266	–	–	–
KL/H	0.701	0.037	0.58	93.0%	30.0%
w-CT	0.799	0.003	4.92	92.9%	44.4%
w-KL	0.833	0.001	65.9	94.9%	20.0%
w-KL/H	0.845	< 0.001	0.43	94.8%	40.0%

*SCr* serum creatinine, *CKD-EPI* chronic kidney disease epidemiology collaboration, *CT* cortical thickness, *KL* kidney length, *KL/H* kidney length/height ratio, *w* weighted for cortical echogenicity factor

## Discussion

The exponentially increasing burden of chronic kidney disease is now recognized as a worldwide public health problem. Therefore, efforts to improve detection, treatment and monitoring of clinical outcomes would be valuable. For practical reasons, several serum creatinine-based formulas for predicting GFR were created with the CKD-EPI equation, which is currently considered the most accurate GFR estimation tool [12].

The most important finding of our study was the improvement in the accuracy of detecting renal dysfunction and histological changes using traditional ultrasound parameters if cortical echogenicity is taken into account. Moreover, w-KL/H performed better than w-KL or w-CT. Although the AUROC for w-KL/H was higher than w-CT and slightly higher than w-KL, the significance of the difference between them was not checked statistically. However, an AUROC of > 0.7 to < 0.8 is considered acceptable while > 0.8 to < 0.9 is considered excellent [15].

Renal length, measured by sonography, is the most commonly used surrogate marker to predict renal function. Since renal length correlates well with body height [16], certain authors advocate the use of relative instead of absolute renal length to overcome sex and height differences [10]. In accordance with this, our data show that relative instead of absolute renal length more accurately predicted renal dysfunction. KL/H is easy to calculate and it is reasonable to believe that is highly reproducible too.

For renal medical disease purposes, the assessment of renal parenchymal hyperechogenicity is also important [17]. Cortical echogenicity depends upon backscatter echo height yielded from interfaces with glomeruli, tubules and the interstitium [9]. Glomeruli account for only 8% of the renal volume and seem to play a less important role in cortical echogenicity [7]. Indeed, increased cortical echogenicity correlates better with the severity and chronicity of tubulointerstitial changes than glomerular changes [5]. It is also important to keep in mind that, among renal histological changes, interstitial fibrosis and tubular atrophy correlate better with renal function than glomerular changes [18]. Taken together, it is highly plausible that ultrasound parameters weighted towards cortical echogenicity could better detect renal dysfunction and histological changes than their raw counterparts.

In this study, KL, the KL/H ratio and cortical thickness were weighted towards renal cortical echogenicity to build new indexes. Numerical values were assigned to qualitative renal cortical echogenicity evaluation. The rationale for the assignment of semi-quantitative arbitrary values for renal cortical echogenicity relative to the liver is based on the mean pixel density (MPD) ratio of their echogenicities. If both echogenicities are equal, the MPD ratio is 1. When cortical renal echogenicity is less than liver MPD, the ratio is less than 1, and when cortical echogenicity is higher than the liver, the MPD is higher than 1. Indeed, in normal controls, the MPD ratio range is between 0.812 and 0.987 [19], while in chronic kidney disease patients with increased renal cortical echogenicity, a value of 1.15 has been reported [20]. In a study carried out on patients with parenchymal renal disease confirmed by biopsy, the MPD ratio ranged from 0.71 to 1.89 [13]. Accordingly, it is reasonable to assign factors of 1.17, 1.00 and 0.69 for a qualitative evaluation of renal cortical echogenicity less than, equal to and greater than that of the liver, respectively. These values were used as weighting factors for KL, the KL/H ratio and cortical thickness to build composite indexes taking renal cortical echogenicity into account.

In accordance with this line of reasoning, w-KL/H was able to predict increased serum creatinine and GFR < 45 ml/min estimated by means of CKD-EPI and had even better performance in distinguishing glomerular sclerosis, glomerular obsolescence and interstitial changes than any of the other parameters used in this study.

One reason for the better performance of parameters after weighing for cortical echogenicity relies on the spread of data values. Indeed, there was little change in the mean of every parameter studied; however, the standard deviations increased as much as two-fold. As a consequence, this increase in the dispersion of values made the variable more suitable to mirror the large variation in renal function and extension of histological changes seen in CKD patients.

The referral of a patient for kidney biopsy is based on the assumption that the finding of reversal changes would be valuable for guiding therapy. Therefore, a test able to obviate unnecessary biopsies in severe chronic changes patients would be useful. Since the most important point in the contraindication of biopsy is to be sure that the patient has severe chronic changes, a test with high sensitivity is preferable than a highly specific one. In accordance with, the cut-off was chosen based on an approach to clinical decision making [14] for a sensitivity as close as possible to 95%. A cut-off value for w-KL/H equal or less than 0.43 had a sensitivity of 94.8% to predict severe chronic changes, which could be helpful to refine the decision-making process in doubtful cases of renal biopsy indication. The extreme cut-off values of SCr and CKD-EPI make them unsuitable as clinical tools and crude cortical thickness and kidney length did not have any predictive power in the present study.

A potential limitation of our study is that cortical echogenicity was evaluated visually rather than quantitatively. A criticism of the visual evaluation of renal cortical echogenicity relative to the liver is that some kidneys slightly less or slightly more echogenic than the liver might be interpreted as equal to the liver, a caveat regarding accuracy. However, it is unlikely that a kidney less echogenic than the liver could be interpret as higher. Therefore, cases that account for the smallest and largest values are never misinterpreted. Second, variables that could affect renal cortical echogenicity, like the subject’s state of hydration [19] or the administration of furosemide [21] were neglected. However, water loading determined a mean increase in echogenicity of only 6.4% [19] and the qualitatively furosemide effect was limited to the medullary pyramids [21].

Although the assignment of an individual weighting echogenicity factor to an individual patient instead of a mean for each subset of patient seems more appropriate, it is difficult to integrate into clinical practice because it is cumbersome. Therefore, the use of only three different weighting factors renders the index more useful as a clinical tool. Moreover, the index uses factors that can be easily obtained in clinical practice (patient height) and in kidney ultrasound studies (cortical echogenicity and kidney length).

In summary, the data obtained in this study show that KL/H weighted towards cortical renal echogenicity exceeded the other parameters as a marker of renal impairment and severe chronic histological changes in CKD patients. Therefore, reporting renal cortical echogenicity on a semi-quantitative scale applied as a weighting index to simple kidney measurements obtained at conventional renal ultrasound examination can be useful in detecting meaningful losses of renal function.

## Conclusion

The newly derived w-KL/H index bears a close relationship to the degree of chronic glomerular and acute and chronic interstitial changes in patients referred for renal biopsy. In accordance with this, routine calculation of the w-KL/H index may be of help as a non-invasive triage tool to identify patients with significant renal disease. In addition, it might be useful to guide therapeutic decisions.

## Data Availability

The datasets used and/or analysed during the current study are available from the corresponding author on reasonable request. The requirement should first be submitted to ethical committee.

## Abbreviations

CKD: Chronic kidney disease
CKD-EPI: Chronic Kidney Disease Epidemiology Collaboration
CNF: Crescent and fibrinoid necrosis
CT: Cortical thickness
eGFR: estimated glomerular filtration rate
GC: Glomerular cellularity
GO: Glomerular obsolescence
GS: Glomerular sclerosis
IF: Interstitial fibrosis
II: Interstitial infiltrate
KL: Kidney length
KL/H: Kidney length/body height ratio
MP: Mesangial proliferation
MPD: Mean pixel density
SCr: Serum creatinine
TA: Tubular atrophy
w: derived cortical echogenicity-weighting factor
